# Metabolite Profiling of Barley Grains Subjected to Water Stress: To Explain the Genotypic Difference in Drought-Induced Impacts on Malting Quality

**DOI:** 10.3389/fpls.2017.01547

**Published:** 2017-09-07

**Authors:** Xiaojian Wu, Kangfeng Cai, Guoping Zhang, Fanrong Zeng

**Affiliations:** ^1^Agronomy Department, Zhejiang University Hangzhou, China; ^2^Zhejiang Academy of Agricultural Sciences Hangzhou, China

**Keywords:** barley (*Hordeum vulgare* L.), metabolite profiling, β-amylase activity, β-glucan content, water stress

## Abstract

Grain weight and protein content will be reduced and increased, respectively, when barley is subjected to water stress after anthesis, consequently deteriorating the malt quality. However, such adverse impact of water stress differs greatly among barley genotypes. In this study, two Tibetan wild barley accessions and two cultivated varieties differing in water stress tolerance were used to investigate the genotypic difference in metabolic profiles during grain-filling stage under drought condition. Totally, 71 differently accumulated metabolites were identified, including organic acids, amino acids/amines, and sugars/sugar alcohols. Their relative contents were significantly affected by water stress for all genotypes and differed distinctly between the wild and cultivated barleys. The principal component analysis of metabolites indicated that the Tibetan wild barley XZ147 possessed a unique response to water stress. When subjected to water stress, the wild barley XZ147 showed the most increase of β-amylase activity among the four genotypes, as a result of its higher lysine content, less indole-3-acetic acid (IAA) biosynthesis, more stable H_2_O_2_ homeostasis, and more up-regulation of *BMY1* gene. On the other hand, XZ147 had the most reduction of β-glucan content under water stress than the other genotypes, which could be explained by the faster grain filling process and the less expression of β-glucan synthase gene *GSL7*. All these results indicated a great potential for XZ147 in barley breeding for improving water stress tolerance.

## Introduction

Global warming and climate change have become a primary concern worldwide (Intergovernmental Panel on Climate Change [IPCC], 2012). Since the middle of the 20^th^ century, there have been considerable changes in the nature of droughts, extreme weather events, and floods in many regions of the world, which caused marked damage to crop production and great threat to global food security (Wheeler and Von Braun, 2013; Lesk et al., 2016). It has been estimated that, during 1964–2007, droughts significantly reduced cereal production by 10% on average, and this percentage was increasing annually, due to the rising drought severity, increasing vulnerability, and exposure to drought (Lesk et al., 2016). In recent two decades, the effect of drought stress on crop growth, yield, and quality was increasingly becoming a major issue of scientific concerns (reviewed by Kang et al., 2009; Alqudah et al., 2010). And, breeding programs with the aim to enhance crop productivity under drought stress are a top priority in the era of climate change (Jagadish et al., 2015). The occurrence of water stress at the reproductive stage is the most critical, as it strongly impacts yield and seed quality. Understanding the response mechanism of drought stress at crop reproductive stage will help to partially address some of concerns for improving crop tolerance to drought stress and to minimize consequent impacts.

Barley is the fourth most important cereal crop worldwide in terms of planting area, and is primarily used for food, brewing, and animal feed (Druka et al., 2011). In last few decades, malt quality of barley grains was heavily addressed and found to be associated with many chemical constituents and enzymes, such as β-amylase activity and β-glucan content (Wei et al., 2009a). β-Amylase, which is a key factor affecting the capacity of starch degradation during grain germination (Beck and Ziegler, 1989), is closely related to diastatic power (DP) (Liu et al., 2005). There have two genes encoding β-amylase been identified: *BMY1* and *BMY2*. Of them, *BMY1* is mainly expressed during the gain filling stages, and plays a vital role in regulating the gain β-amylase activity and malting quality (Li et al., 2002). And, low β-glucan content is required for brewing as it favors high wort filtration rate and malt extract. In barley grains, the β-glucan content was found to be largely regulated by three genes, *GSL1*, *GSL4*, and *GSL7* (Schober et al., 2009). Furthermore, it has been documented that both β-glucan content and β-amylase activity are not only genetically controlled, but also greatly affected by environmental factors (Zhang et al., 2001, 2006; Djukić and Knežević, 2014; Rakszegi et al., 2014). It was reported that β-glucan content in barley grains was highly reduced when plants suffered from heat or water stress during grain-filling process (Macnicol et al., 1993; Christensen and Scheller, 2012). On the other hand, total protein content in barley grains was generally increased under the drought or heat stress condition, consequently resulting in higher β-amylase activity (Todaka et al., 2000; Yin et al., 2002; Qi et al., 2006). Such reduction of β-glucan content and increase of β-amylase activity were also observed in our previous study on water stress at the grain-filling stage, and they were closely associated with the reduction of grain yield and increase of protein content, which would consequently cause deterioration and instability of grain yield and malt quality (Wu et al., 2015). Furthermore, our previous study also found such effect of water stress on the β-glucan content and β-amylase activity varied dramatically among barley genotypes, especially between the Tibetan wild barley and cultivated ones (Wu et al., 2015). Therefore, to achieve the stability of grain yield and malt quality under water stress condition, it is imperative to reveal the mechanisms in the adverse impact of water stress on malt quality, including β-glucan content and β-amylase activity, and the difference among barley genotypes in the response to water stress.

Metabolite profiling may provide a comprehensive approach to assess a broader spectrum of constituent analysis and has been proven to be a suitable tool for investigating the metabolites changes caused by genetic modification and environmental condition (Röhlig et al., 2009; Frank et al., 2011; Cañas et al., 2015). Currently, metabolomic analysis has been extensively conducted on the changes of metabolite profiles under water stress in many species, such as tomato (Rivero et al., 2015), soybean (Silvente et al., 2012), maize (Sun et al., 2015), and rice (Li et al., 2015). In barley, several metabolite profiling studies have also been performed to determine the influence of water stress on free amino acids (Lanzinger et al., 2015), abscisic acid, and the oxidative status (Thameur et al., 2014). However, to our knowledge, no such report has been addressed to the effect of water stress on malt quality. In the present study, four wild and cultivated barley genotypes contrasting in drought tolerance were used to compare metabolic changes in the response to water stress using gas chromatography-mass spectrometry (GC-MS). The objectives of the current work are to determine the mechanisms in the influence of water stress on β-amylase activity and β-glucan content, and to reveal the reasons why barley genotypes differ in their responses to water stress.

## Materials and Methods

### Plant Materials and Sampling

Four barley genotypes contrasting in drought tolerance were used in this study based on our previous research (Wu et al., 2015). Of them, XZ5 (sensitive to drought) and XZ147 (tolerant to drought) are the Tibetan wild barley accessions, while Triumph (Tr) is a cultivar and TL43 is an ABA-insensitive mutant derived from Tr (Romagosa et al., 2001). Ten seeds of each barley genotype were sown in a pot with 7.5 kg (7 l) sandy-clay soil in mid-November 2015 at Zijin’gang Campus, Zhejiang University, Hangzhou, China. Three weeks later, only five uniform healthy barley seedlings were preserved for the experiment. During the growth of barley seedlings, all the pots were well irrigated to the water content of 40% (equaling to water potential of -0.15 MPa). When half the plants of each barley genotype were at anthesis stage in March 2016, the spikes at the same stage were tagged for further measurements, and the water stress treatment (drought, abbreviation of T) was conducted subsequently by stopping water supply to make the soil water content drop to be around 14% (equaling to water potential of -0.75 MPa, taking approximately 3 days, Supplementary Figure S1) and maintained for another 14 days. Thereafter, the water supply was resumed to control level. For control condition (normal water level, abbreviation of CK), the water content in soil was continuously maintained around 40% (equaling to water potential of -0.15 MPa). During the water stress treatment, all the pots were irrigated everyday by a repeated watering-testing procedure to get the required soil water content (40% for the control and 14% for drought). For each time, 20 mL water was added to each pot without disturbing the plants and 10 min later soil water content was tested using HH2 Moisture Meter (Delta-T Devices, Cambridge, United Kingdom). Once the soil water content reached to the required value, the watering was stopped.

At the 3^rd^ (3d) and 7^th^ (7d) of water stress treatment, the tagged spikes of each genotype were collected from both control and water stress treatment, and immediately stored at -80°C for the subsequent analysis. At maturity, the spikes on main stems were harvested and oven-dried at 37°C. Oven-dried grains were ground with a cyclone mill (SCINO CT410, FOSS) equipped with a 0.5 mm sieve and the flour samples were stored at -20°C until to be analyzed.

### Measurements of β-Amylase Activity and β-Glucan Content

β-Amylase activity was measured using a Betamyl Assay Kit (Megazyme International, Ireland Ltd.) according to McCleary and Codd’s (1989) method. Total β-glucan content was assayed using a commercial kit (Megazyme International, Ireland Ltd.) according to McCleary and Codd’s (1991) method.

### Metabolite Profiling

Metabolites of barley grains were extracted according to Lisec et al. (2006) with some modification. In brief, finely grinded barley powder (approx. 100 mg) was mixed well with 1,400 μl of 100% methanol (pre-cooled at -20°C) and 60 μl of ribitol (0.2 mg/ml stock in dH_2_O, as an internal quantitative standard) in 2 ml centrifuge tube, and then placed in a shaking bath at 70°C for 10 min and centrifuged at 11,000 × *g* for 10 min. The supernatant was collected into a 10 ml centrifuge tube containing 750 μl chloroform (pre-cooled at -20°C) and 1,500 μl deionized water (4°C), vortexed for 30 s, and then centrifuged at 2,200 × *g* for 15 min. One hundred and fifty microliters of supernatant was collected into a new 1.5 ml centrifuge tube and vacuum-dried for 1 h. Thereafter, 40 μl methoxyamine pyridine solution (20 mg/ml) was added into the tube, incubated in a shaker at 37°C for 2 h, then 70 μl MSTFA reagent was added, and shaked for 30 min at 37°C. Metabolites contents were subsequently determined using Agilent 6890N GC/5975B MSD (Agilent, United States). The program of temperature rise was set as: initial temperature of 70°C for 2 min, 10°C/min rate up to 140°C, 4°C/min rate up to 240°C, 10°C/min rate up to 300°C, and then staying for 8 min.

The raw signals were imported into software AMDIS (Version 2.71) to search for metabolites from its default universal database. The total mass of signal integration area was normalized for each sample, with the total integral area of each sample being normalized to 10,00,000. Finally, the normalized data were imported into MetaboAnalyst online analysis software^1^, employing PLS-DA model and the first principal component of VIP (variable importance in the projection) values (VIP.1) combined with Student’s *t*-test (*T*-test) (*p*, 0.01), to find differentially accumulated metabolites (Lisec et al., 2006).

### Quantitative Real-time PCR

The relative transcript level of the genes encoding β-amylase and β-glucan synthases was determined through quantitative real-time PCR (qRT-PCR). Total RNAs in barley grains were extracted using a RNA plant Plus Reagent Kit [Tiangen Biotech (Beijing) Co. Ltd.] according to the instruction. RNA was reverse-transcripted to cDNAs using a PrimeScript RTF Reagent Kit with gDNA Eraser (Takara Bio Inc.), and then stored at -20°C for subsequent PCR analysis. The qRT-PCR was performed on CFX96 Touch^TM^ Real-Time PCR Detection System (Bio-Rad Laboratories, Inc.) with an iTaq Universal SYBR Green Supermix (Bio-Rad Laboratories, Inc.). Primer sequences for qRT-PCR analysis were listed in Supplementary Table S1.

### Data Analysis

Prior to data analysis, quantitative normalization within replicates was transformed by logarithmic base of 2. MetaboAnalyst online analysis software^2^ was used to build heatmap diagram (Xia and Wishart, 2011). Meanwhile, hierarchical cluster analysis (HCA) and principal component analysis (PCA) models were performed for all samples. The different significance of metabolites between treatment and control or among genotypes was tested using *T*-test and ANOVA analysis on SPSS 20.0 software.

## Results

### Effect of Water Stress on Grain Weight, β-Amylase Activity, and β-Glucan Content

**FIGURE 1:**
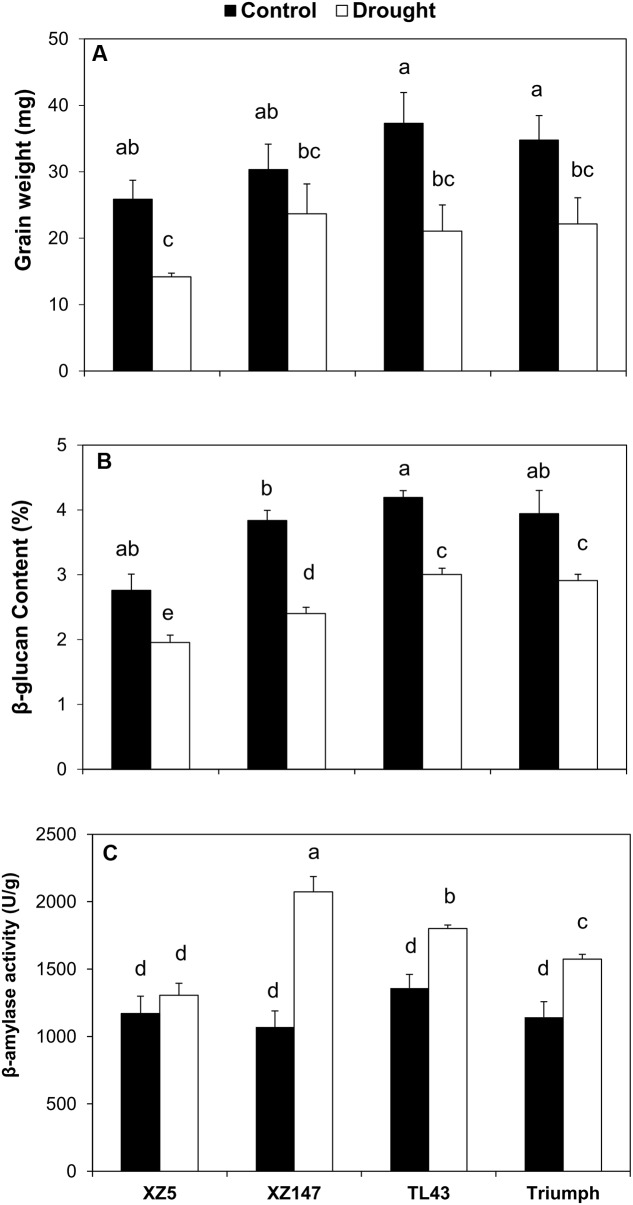
The effect of water stress on grain weight **(A)**, β-amylase activity **(B)**, and β-glucan content **(C)** of different barley genotypes. Different letters indicate the significant difference between each genotype × treatment combination at 95% probability. Data are mean ± SD.

Drought stress dramatically reduced grain weight of barley genotypes Tr, TL43, and XZ5 with XZ5 showing the most reduction, whereas no significant effect of drought on grain weight was observed for XZ147 (**Figure 1A**). Similarly, drought stress caused a significant decrease in β-glucan content of barley grains, in particular for XZ147 (37.38%) (**Figure 1B**). On the other hand, β-amylase activity was remarkably increased in XZ147, TL43, and Tr by 93.76, 37.57, and 32.55% under water stress compared with the control, but only a tiny increase (11.52%) in β-amylase activity was seen for XZ5 (**Figure 1C**).

### Changes of Metabolites in the Response to Water Stress

**FIGURE 2:**
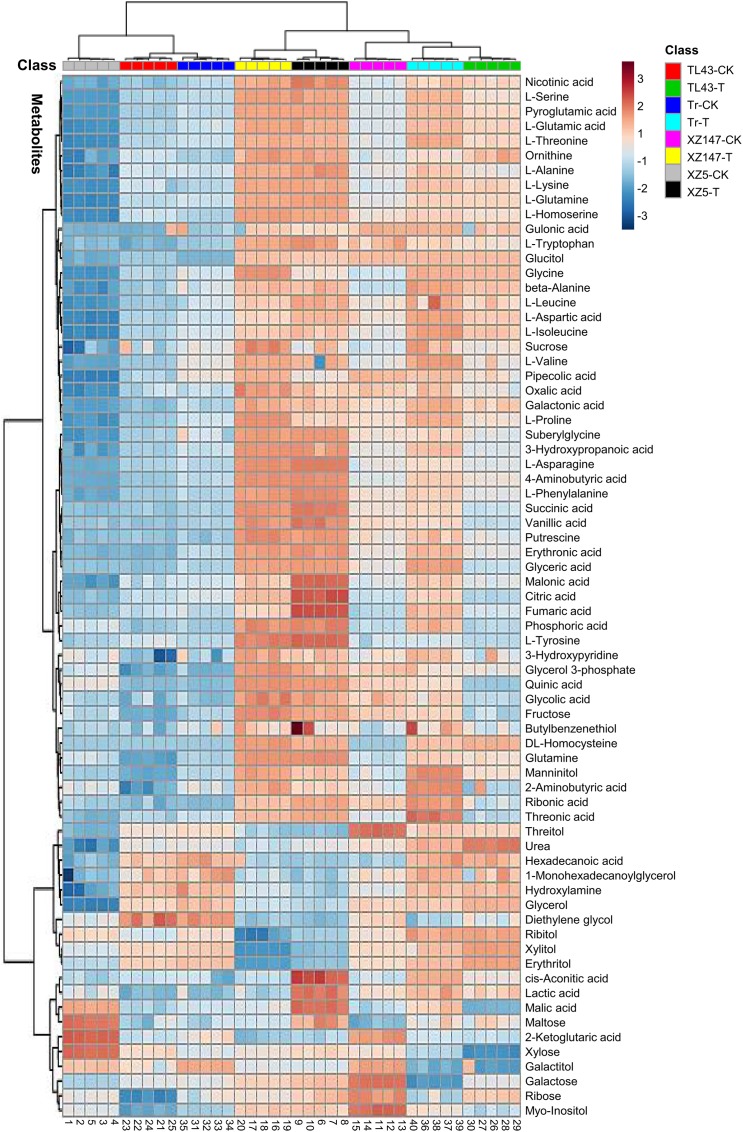
Hierarchical cluster analysis of 71 metabolites and 8 genotype × treatment combinations (distance measure using Euclidean and clustering algorithm using Ward.D). CK, control condition with soil water content of 40%; T, water stress treatment with soil water content of 14% and Tr, Triumph. The scale –3 (dark blue, the lowest) to 3 (dark red, the highest) indicated the relative content of each metabolite for each genotype × treatment combination.

**FIGURE 3:**
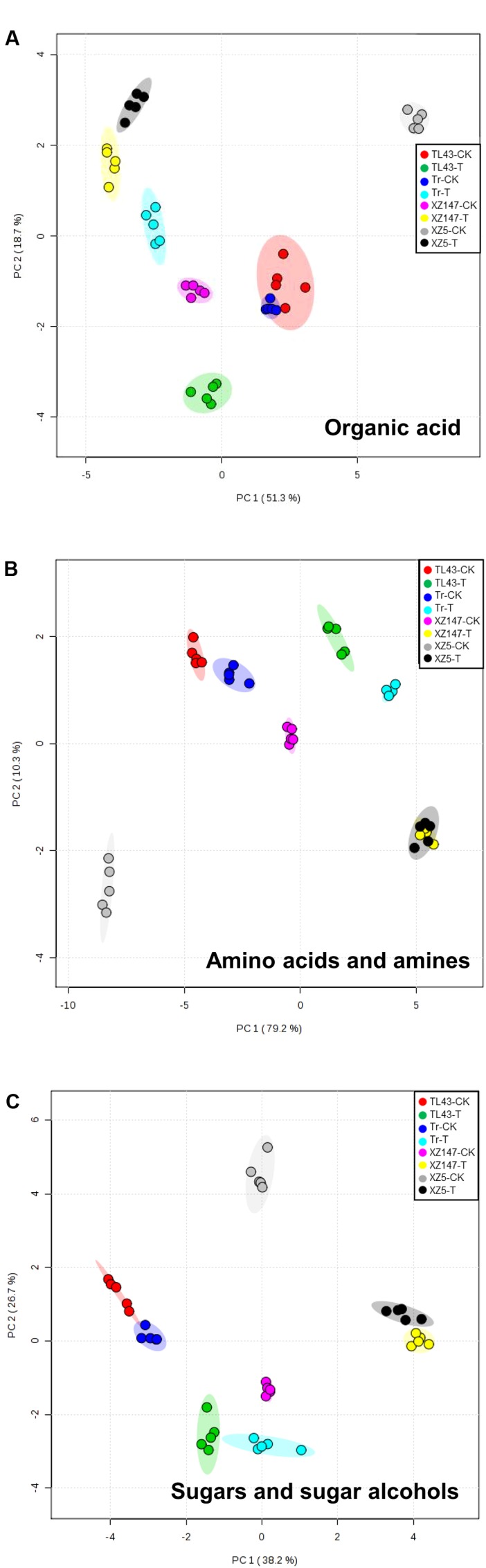
Principal component analysis (PCA) of metabolic profiles in grains of four barley genotypes under the two water treatments. **(A)** PCA of fraction I, organic acid; **(B)** PCA of fraction II, amino acids and amines; and **(C)** PCA of fraction III, sugars and sugar alcohols. CK, control condition with soil water content of 40%; T, water stress treatment with soil water content of 14%; Tr, Triumph; PC1, the first principal component; and PC2, the second principal component.

In the present study, the metabolic profiles in barley grains at filling stage were investigated. Totally, 71 differently accumulated metabolites were successfully identified, and their relative contents were significantly affected by water stress for all genotypes (**Figure 2**). According to the chemical profile, these metabolites could be classified into three fractions: organic acids (fraction I), amino acids and amines (fraction II), and sugars and alcohols (fraction III). In order to determine the effect of water stress on the metabolites, PCA was conducted separately on each metabolite fraction. The two treatments (water stress and control) and four genotypes were clearly separated by two principal components (**Figure 3**). For organic acid fraction, samples from the control and water stress for XZ5, Tr, and TL43 were clearly separated by PC1, which could explain 51.3% of the total variation, whereas the two treatments for XZ147 were separated by PC2, which only explained 18.7% of the total variation (**Figure 3A**). For fraction of amino acids and amines, PC1 clearly separated the samples of the two water treatments for all barley genotypes, explaining 79.2% of the total variation; and PC2 obviously separated the wild (XZ5 and XZ147) and cultivated genotypes (Tr and TL43) under the condition of water stress, explaining 10.3% of the total variation (**Figure 3B**). Furthermore, PCAs based on the metabolite data of sugars and alcohols (fraction III) showed a strong influence of water stress, and could explain 38.2 and 26.7% of the total variation, respectively (**Figure 3C**).

**FIGURE 4:**
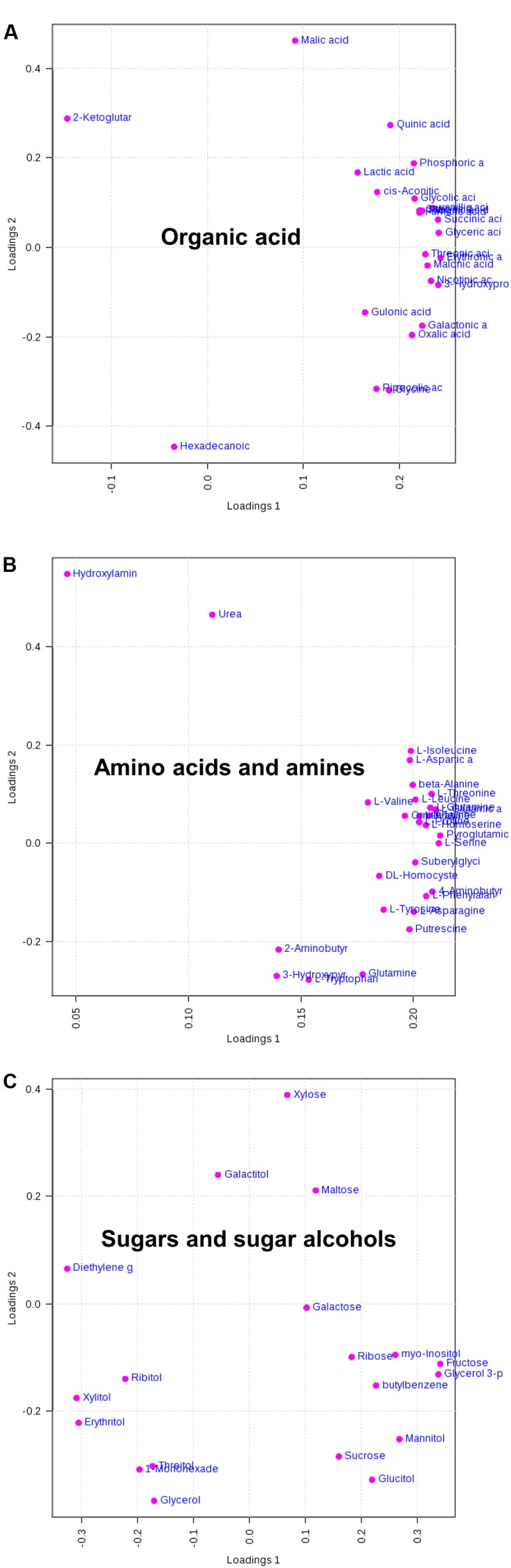
The corresponding loading plots of metabolite profiling data from fraction I (organic acid, **A**), fraction II (amino acids and amines, **B**), and fraction III (sugars and sugar alcohols, **C**) between PC1 and PC2.

The loading plots revealed that constituents from all identified substance classes (organic acids, amino acids and amines, and sugars and alcohols) were responsible for the variation (**Figure 4**). Fraction I (acids) and fraction II (amino acids and amines) were the major prominent factors for shifting on PC1 and PC2 (**Figures 4A,B**). The loading plots of fraction III (sugars and alcohols) showed that monosaccharides, including fructose, sucrose, and maltose, as well as alcohol and mannitol were obviously increased under water stress (**Figure 4C** and **Table 1**). Furthermore, the majority of metabolites in fractions I and II were also affected by water stress (**Figures 4A,B** and **Table 1**).

**Table 1:** Relative concentration and the fold changes of the major metabolites in the grains of the different barley genotypes under water stress.

Metabolites	Relative concentration of the major metabolites								Fold change			
		
	XZ5		XZ147		TL43		Triumph		log_2_^(drought/control)^			
					
	Control	Drought	Control	Drought	Control	Drought	Control	Drought	XZ5	XZ147	TL43	Triumph
Maltose	0.19	0.14	0.03	0.05	0.04	0.07	0.04	0.07	-0.48**	0.85	0.81	0.78
Fructose	14.78	45.17	32.96	55.54	8.16	13.90	13.17	30.28	1.61**	0.75**	0.77**	1.20**
Sucrose	3.27	4.41	3.76	5.42	3.79	4.15	3.98	4.93	0.43**	0.53**	0.13	0.31**
Mannitol	8.41	29.96	13.34	42.27	5.07	21.29	10.02	52.06	1.83**	1.66**	2.07**	2.38**
Myo-inositol	13.25	16.03	21.23	16.04	9.43	11.91	11.10	14.47	0.27**	-0.40**	0.34**	0.38**
Xylitol	9.04	3.17	19.86	1.47	20.94	48.95	25.41	27.47	-1.51	-3.75**	1.22**	0.11
Xylose	0.90	0.32	0.25	0.06	0.28	0.04	0.16	0.13	-1.49**	-2.20**	-2.65**	-0.28
Citric acid	4.46	26.98	6.65	13.95	6.32	5.78	7.63	11.84	2.60**	1.07**	-0.13	0.63**
Fumaric acid	0.49	2.69	0.62	1.18	0.68	0.76	0.67	1.42	2.44**	0.92**	0.15	1.08**
2-Ketoglutaric acid	4.66	1.72	3.69	1.48	1.87	1.85	2.44	1.98	-1.44**	-1.32**	-0.02	-0.30**
Malic acid	68.12	77.74	48.14	52.43	46.09	40.97	50.07	61.86	0.19**	0.12**	-0.17**	0.31**
Succinic acid	1.36	5.24	2.56	4.59	1.47	1.93	1.62	3.06	1.95**	0.84**	0.40**	0.92**
Threonic acid	0.20	1.12	0.45	0.92	0.29	0.31	0.36	1.69	2.50**	1.04**	0.09	2.24**
beta-Alanine	0.21	1.04	0.46	1.04	0.36	0.97	0.56	1.25	2.28**	1.17**	1.41**	1.16**
L-Asparagine	20.69	294.18	69.48	239.50	37.40	65.04	42.64	99.10	3.83**	1.79**	0.80**	1.22**
L-Glutamic acid	1.22	14.42	4.97	15.00	3.01	9.37	4.27	12.25	3.57**	1.59**	1.64**	1.52**
L-Glutamine	0.57	43.67	8.01	36.62	3.83	17.01	4.92	18.25	6.27**	2.19**	2.15**	1.89**
L-Glysine	0.77	17.00	4.32	17.03	3.29	9.37	2.58	11.81	4.47**	1.98**	1.51**	2.19**
L-Isoleucine	0.61	3.37	2.08	3.17	1.35	3.30	1.82	4.51	2.46**	0.61**	1.29**	1.31**
L-Leucine	0.49	3.47	1.84	2.58	0.86	2.40	1.27	3.65	2.84**	0.48	1.48**	1.52**
L-Lysine	5.37	15.20	4.46	17.03	3.63	9.37	2.58	11.81	1.50**	1.93**	1.37**	2.19**
L-Proline	6.98	43.17	36.81	82.47	13.08	33.06	21.30	63.10	2.63**	1.16**	1.34**	1.57**
L-Serine	0.84	14.25	3.75	14.84	2.12	5.78	2.63	11.13	4.09**	1.98**	1.45**	2.08**
L-Threonine	1.17	8.07	3.80	8.52	2.73	5.89	3.20	8.85	2.79**	1.16**	1.11**	1.47**
L-Valine	1.42	8.59	4.66	11.47	2.34	5.85	4.93	12.62	2.60**	1.30**	1.32*	1.36**
Putrescine	0.13	0.72	0.34	0.80	0.14	0.27	0.15	0.50	2.45**	1.22**	0.94**	1.70**
Pyroglutamic acid	3.55	87.87	20.49	87.42	9.27	39.87	13.30	63.41	4.63**	2.09**	2.10**	2.25**


The relative contents and fold changes [calculated using formula: log_2_ (drought/control)] of the 25 dominant metabolites in grains were listed in **Table 1**. Among them, the relative contents of 19 metabolites were increased by water stress for all barley genotypes, including several osmoprotectants like mannitol and L-proline (**Table 1**). However, such water stress-induced increase in these metabolites showed an obvious genotypic difference, with the wild barley XZ5 showing the most increase in these metabolites except mannitol. On the other hand, the relative content of 2-ketoglutaric acid was reduced in all genotypes except TL43 under water stress, and the reduced extent differed dramatically between the two wild barleys (-1.44-fold in XZ5 and -1.32-fold in XZ147) and the cultivar Tr (-0.30-fold) (**Table 1**). Furthermore, there were five metabolites showing different changes in response to water stress between the four barley genotypes. For instance, water stress significantly reduced the relative content of maltose in XZ5 by -0.48-fold, but no significant change in it was seen for the other three genotypes. The effect of water stress on myo-inositol content differed among barley genotypes, with XZ147 being reduced (-0.40-fold); and XZ5, TL43, and Tr being increased (0.27-, 0.34-, and 0.38-fold, respectively). Similarly, water stress reduced xylitol content in wild barleys (-1.51-fold in XZ5 and -3.75-fold in XZ147) but increased it in the cultivated genotype TL43 (1.22-fold), and caused little change in Tr (0.11-fold).

### Effect of Water Stress on Metabolic Pathway

**FIGURE 5:**
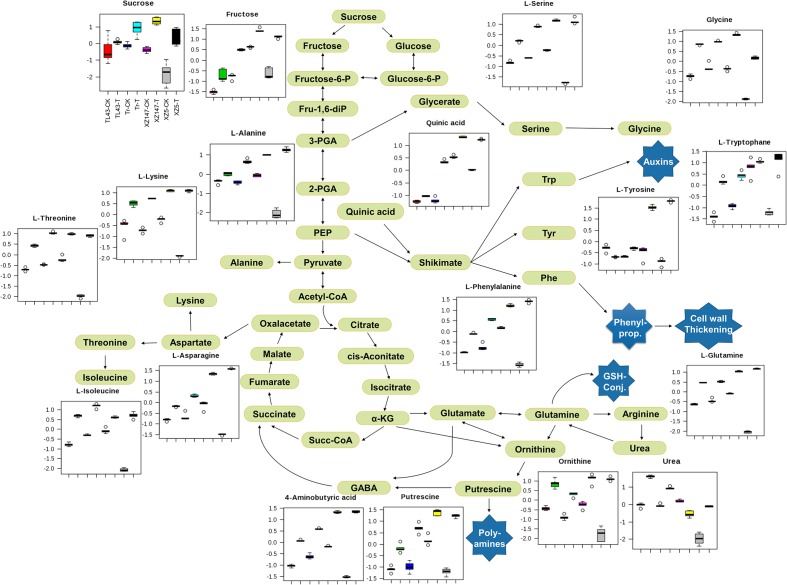
Schematic overview of metabolic pathways in response to water treatments for different barley genotypes. CK, control condition with soil water content of 40%; T, water stress treatment with soil water content of 14%; and Tr, Triumph. Black arrows indicate specific metabolic steps.

The metabolites with significant changes under drought stress were illustrated on the metabolic pathway (**Figure 5**). The elevated level of sucrose and TCA cycle components (citric acid, succinic acid, fumaric acid, malic acid) probably indicated the enhanced energy metabolism (Warth et al., 2015) and elevated respiratory rates (Bolton, 2009) for plant defense actions, such as drought stress protective program (**Figures 2**, **5**). Along with the increase of intermediates in TCA cycle, several amino acids displayed increased level at different extent in response to drought stress, which is always associated with different plant defense mechanisms (**Figures 2**, **5**; Warth et al., 2015). Quinic acid – the precursor of the shikimate pathway showed higher accumulation under drought of all genotypes, especially in wild genotypes. This pathway provides important aromatic secondary metabolites such as phenylpropanoids as well as the plant hormone auxins (**Figure 5**; Sugawara et al., 2009; Zhao, 2010; Mashiguchi et al., 2011). In mitochondrion of plants, arginine is transferred to ornithine and urea by arginase, which is crucial for the mobilization of nitrogen (Witte, 2011). In our study, surprisingly, only in wild barley XZ147 the urea biosynthesis was inhibited, indicating that XZ147 processes a unique nitrogen metabolic way different from the other genotypes (**Figure 5**). Furthermore, the enhanced glutamate recycling could significantly maintain GSH levels under abiotic stress (**Figures 2**, **5**; Paulose et al., 2013). These results revealed that drought stress was able to modify both the primary carbohydrate metabolism and the primary nitrogen metabolism, and induce several defense responses as well.

### Effect of Water Stress on Transcriptional Levels of the Genes Encoding β-Amylase and β-Glucan Synthases

**FIGURE 6:**
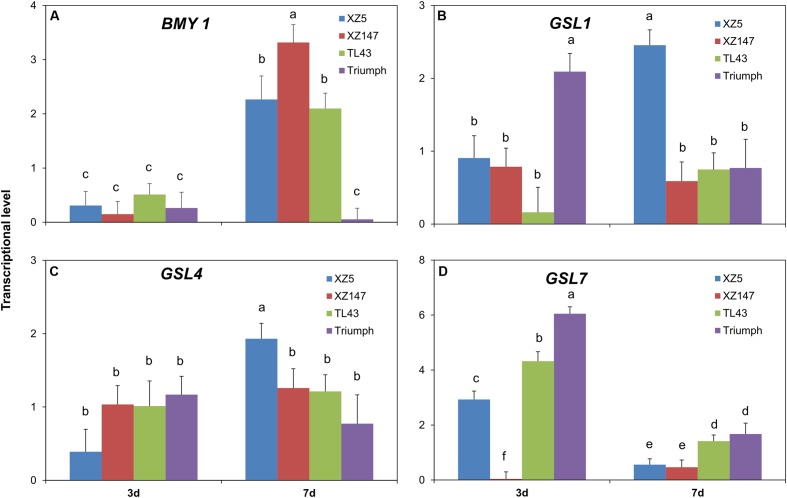
Changes in transcriptional levels of *BMY1* **(A)**, *GSL1* **(B)**, *GSL4* **(C)**, and *GSL7* **(D)** in caryopsis of different barley genotypes after onset of water stress for 3d and 7d. *GSLs*, β-glucan synthase genes and *BMY1*, β-amylase gene 1. Different letters indicate the significant difference at 95% probability. Data are mean ± SD.

The effect of water stress on the expression of genes encoding β-amylase and β-glucan synthesis was presented in **Figure 6**. It could be seen that the response of the expression of four examined genes to water stress was highly duration- and genotype-dependent. At 3d of water stress, transcriptional level of *BMY1* gene (encoding β-amylase) was little affected. However, at 7d, expression of *BMY1* was distinctly increased in XZ5 (2.3-fold), XZ147 (3.3-fold), and TL43 (2.1-fold), but no significant change was observed for Tr (**Figure 6A**) in comparison with control. The transcriptional levels of the three genes encoding β-glucan synthase, *GSL1*, *GSL4*, and *GSL7*, differed greatly among genotypes under water stress. The effect of water stress on expressional patterns of *GSL1* and *GSL4* was quite similar (**Figures 6B,C**). At 3d of water stress, only Tr increased the expression of *GSL1* by 2.1-fold, while at 7d, the expression of both *GSL1* and *GSL4* in XZ5 was increased by 2.5- and 1.9-fold, respectively. The effect of water stress on *GSL7* expression varied greatly over the time of water stress. At 3d of water stress treatment, the expression of *GSL7* was significantly increased in XZ5 (2.9-fold), TL43 (4.3-fold), and Tr (6.0-fold) but dramatically decreased in XZ147 (only 3.8% of the control) (**Figure 6D**). With the exposure time increased to 7d, the water stress caused up-regulation of *GSL7* in XZ5, TL43, and Tr and the down-regulation of *GSL7* in XZ147 were both reduced (**Figure 6D**). In addition, it was easily found that the expression level of *GSL7* was much lower in wild barley than in cultivated ones (**Figure 6D**), being coincide with the results of the β-glucan content (**Figure 1B**).

## Discussion

It is well documented that metabolic changes happen in the plants exposed to water stress, resulting in yield loss (Boyer, 1982; Bray et al., 2000). In this study, water stress induced a significant reduction of grain weight for all genotypes, compared with the control (**Figure 1**), being consistent with the results obtained in our previous work (Wu et al., 2015). However, such reduction of grain weight differed greatly between genotypes, with XZ147 being the least and XZ5 being the most. It was reported that carbohydrates and starch account for 78–83 (MacGregor and Fincher, 1993) and 50–70% (Henry, 1988) of barley grain weight, respectively. Photosynthetic product (glucose) is transported to grains in the form of sucrose, and used for synthesis of starch as well as β-glucan. The inhibited synthesis of starch by water stress may be a major reason for the reduction of grain weight (Chaves, 1991). In this study, the available sucrose transported to grains was dramatically increased under water stress for all barley genotypes, with XZ147 having the most increase (**Figures 2**, **5**). Thanks to its much quicker grain-filling process under water stress (Wu et al., 2017), XZ147 remained relatively smaller change in grain weight than other genotypes (**Figure 1A**).

In the present study, the PCA revealed that the clustering between barley genotypes strongly depended on fraction II (amino acids and amines) and fraction III (sugars and sugar alcohols) (**Figures 4B,C**). So, it can be assumed that these low molecular compounds (amino acids, amines, sugars, and sugar alcohols) played an important role in barley’s tolerance to water stress. Indeed, both our and numerous previous studies have found that the content of amino acids in plant vegetative tissues and reproductive grains changed remarkably when subjected to water stress (Widodo et al., 2009; Sicher et al., 2012; Nam et al., 2014; **Table 1**). It is well known that osmotic adjustment by the accumulation of compatible solutes is a key mechanism for maintaining cell turgor under water stress (Serraj and Sinclair, 2002; Hummel et al., 2010). Proline is one of the main osmoprotectant in plants when subjected to osmotic stress, such as drought or salinity (Delauney and Verma, 1993; Liu and Zhu, 1997). It has been also reported that the accumulation of mannitol and inositol increased in plants when exposed to osmotic stress like salinity (Abebe et al., 2003; Sanchez et al., 2008). In this study, the contents of both proline and mannitol were increased by water stress in all used barley genotypes, whereas the drought tolerant wild barley XZ147 showed the least increase among all genotypes (**Table 1**). Moreover, XZ147 showed the reduction in inositol content under water stress relative to the control, while other three genotypes showed the increase of this metabolite. Very similar results were also obtained in the study on the metabolic responses to salt stress of barley (Widodo et al., 2009). These results suggested that the increasing accumulation of osmoprotectants like proline and mannitol is an important adaptive strategy of barley to survive under the terminate drought stress, especially for the sensitive genotypes. Likewise, synthesis of some amino acids and organic acids was significantly enhanced under water stress (**Table 1**), which could be in favor of osmotic adjustment and membrane stability (Ueda et al., 2004; Chen et al., 2007; Widodo et al., 2009). It has been previously documented that poly-amines (PAs) associated with some related amino acids play important roles in the adaptation to abiotic and biotic stresses by regulating carbon/nitrogen homeostasis or acting as signaling molecules and compatible solutes under drought stress (Moschou et al., 2008; Liu et al., 2010; Hussain et al., 2011; Zeier, 2013). However, the biosynthesis of these compatible solutes by plants is always at the high cost of photo-assimilates and energy, which consequently sacrifice the grain yield and quality (Bolton, 2009). In this study, we found a much higher increased accumulation of osmoprotectants (such as proline, mannitol, fructose, sucrose, and citric acid) in drought-sensitive wild XZ5 than drought-tolerant wild XZ147 (**Table 1**), which consequently severely affected the yield formation and the two malt quality traits (β-amylase activity and β-glucan content) (**Figure 1**), completely coinciding with the findings of Serraj and Sinclair (2002).

β-Amylase activity is an important malt quality parameter, which is positively correlated with DP (Ovesná et al., 2012). Furthermore, a number of studies have demonstrated that β-amylase induction could help plants cope with unfavorable growing conditions (Dreier et al., 1995; Nielsen et al., 1997; Todaka et al., 2000; Kaplan and Guy, 2004), possibly by hydrolyzing more starch to maltose to function as a compatible-solute stabilizing factor (Lu and Sharkey, 2004; Kaplan and Guy, 2005). Hejgaard and Boisen (1980) found that the genotypes rich in lysine had higher β-amylase activity because of more serine protease inhibitor (Z protein) and chymotrypsin inhibitors (CI-1 and CI2). In this study, the grain lysine content was dramatically increased by water stress especially for wild genotypes (**Figures 2**, **5**), being consistent with the results reported previously (Macnicol et al., 1993; MacGregor et al., 1994; Wei et al., 2009b). So, it could be assumed that the increased grain lysine content is an important reason for the increased β-amylase activity under water stress. On the other hand, during grain filling stage of cereal crops or ripening stage of fruits, the increased levels of auxins could be observed along with the inhibition of β-amylase activity for starch accumulation (Eeuwens and Schwabe, 1975; Bangerth et al., 1985; Aufhammer et al., 1986; Purgatto et al., 2001). In the present study, we found that the content of L-Tryptophan, the precursor for auxin biosynthesis (Mano and Nemoto, 2012) was extensively increased by water stress for all used barley genotypes except XZ147. This might be one other explanation for the higher β-amylase activity in XZ147 than in the other genotypes under water stress. Abiotic stress like drought stress always causes excess reactive oxygen species (ROS) accumulation, which will result in cellular damage and consequently yield losses. Fortunately, plants have obtained numerous antioxidants to scavenge the excess ROS, for instance GSH and AsA, which play a key role in intracellular ROS level regulation (Apel and Hirt, 2004; Sairam and Tyagi, 2004; Møller et al., 2007). On the other hand, Wei et al. (2009b) reported that exogenous H_2_O_2_ treatment could elevate barley β-amylase activity, suggesting that the enhanced grain H_2_O_2_ concentration would increase β-amylase activity under osmotic stress. However, the increase in GSH concentration might extinguish this effect. In the present study, we found that the precursor of GSH biosynthesis, L-glutamine, was increased by water stress in all used barley genotypes, with XZ5 being significantly higher than the other genotypes. Such over accumulation of GSH in XZ5 would be helpful for the scavenging of ROS, but not benefit for the induction of β-amylase activity (**Figure 1C**). All these results indicated that the induction of barley grain β-amylase activity was highly correlated with the lysine content, indole-3-acetic acid (IAA) biosynthesis, and H_2_O_2_ homeostasis. Furthermore, our results revealed that the expression level of *BMY1*, which encodes β-amylase in barley grains, was dramatically induced in XZ147 after 7 days of water stress being accompanied by higher β-amylase activity (**Figures 1C**, **6A**). Therefore, it can be hypothesized that the reason for the higher grain β-amylase activity in XZ147 than the other genotypes under water stress might be attributed to its higher lysine content, less IAA biosynthesis, more stable H_2_O_2_ homeostasis, and also the higher up-regulation of *BMY1* gene.

β-Glucan content is a critical parameter for malt brewing, with high β-glucan content always resulting in slower wort filtration rate and beer haze (Bamforth, 1982). It has been reported that β-glucan accumulated in barley grains throughout the whole filling stage (Christensen and Scheller, 2012). Thus, it may be understandable that the faster grain filling process causes the lower β-glucan content. Indeed, our results revealed that the grain β-glucan content in wild barley (XZ147 and XZ5) was decreased much more than that in cultivated barley (Tr and TL43) when subjected to water stress (**Figure 1B**), completely coinciding with our previous results that the grain filling process of wild barley (XZ147 and XZ5) was much faster than the cultivated barley (Tr and TL43) after onset of drought stress (Wu et al., 2017). Furthermore, it has been also reported that the synthesis of β-glucan in barley grains was largely controlled by the genes *GSL1*, *GSL4*, and *GSL7* (Schober et al., 2009). In the present study, we found that, in comparison with the cultivated barley, the expression level of *GSL7* in wild barley was much lower and it was reduced much more with the prolongation of water stress (**Figure 6D**), which could account for the lower β-glucan content in the grains of the wild barley (**Figure 1B**).

In summary, water stress remarkably altered grain weight, β-amylase activity, and β-glucan content of the four genotypes used in this study. When subjected to water stress, the wild barley XZ147 showed the most increase of β-amylase activity among the four genotypes which might be attributed to its higher lysine content, less IAA biosynthesis, more stable H_2_O_2_ homeostasis, and more up-regulation of *BMY1* gene. XZ147 also had the most reduction of β-glucan content under water stress than the other genotypes, which could be explained by the faster grain filling process and the less expression of β-glucan synthase gene *GSL7*. Obviously, XZ147 is interesting for malt barley breeders to develop the new cultivars with high water stress tolerance and stable malt quality.

## Author Contributions

FZ and GZ designed the project; XW and KC carried out the experimental work; XW and FZ wrote the paper with contributions from GZ; and all authors read and approved the paper.

## Conflict of Interest Statement

The authors declare that the research was conducted in the absence of any commercial or financial relationships that could be construed as a potential conflict of interest.

## Footnotes

1 www.metaboanalyst.ca/

2 www.metaboanalyst.ca/
