# Supplementary material for: Metabolite Profiling of Barley Grains Subjected to Water Stress: To Explain the Genotypic Difference in Drought-Induced Impacts on Malting Quality
Source: Front Plant Sci. 2017 Sep 7;8:1547. doi: 10.3389/fpls.2017.01547 (PMC5594086; doi:10.3389/fpls.2017.01547)
Supplement: Supplementary file 1 [file Table_1.DOCX]

**Table S1 qPCR primer sequences**

| **Gene** | **Primer sequence (5’-3’, forward/reverse)** |
| --- | --- |
| *GSL1* | TTAGTGGTTTTGGCAGGTTTG/ CGTGTCTGGAAGGTGGAG |
| *GSL4* | GAACAAGGCGAACCAAGAG/ CAAGCGATTAGCGTCCAAGT |
| *GSL7* | CAAGAAATGCAGACAAGGGTA/ CCAAGACTCAATGCCTAAATCAC |
| *BMY1* | CCTGCCACCATGTAATGGAAC/ AGGTTTCTCTGTCACACTCACACAA |
| *GAPDH* | AAGCATGAAGATACAGGGAGTGTG / AAATTTATTCTCGGAAGAGGTTGTACA |

*GSLs*, β-glucan synthase; *Bmy1*, β-amylase gene 1.
