# Supplementary material for: Metabolite Profiling of Barley Grains Subjected to Water Stress: To Explain the Genotypic Difference in Drought-Induced Impacts on Malting Quality
Source: Front Plant Sci. 2017 Sep 7;8:1547. doi: 10.3389/fpls.2017.01547 (PMC5594086; doi:10.3389/fpls.2017.01547)
Supplement: Supplementary file 2 [file Image_1.pdf]

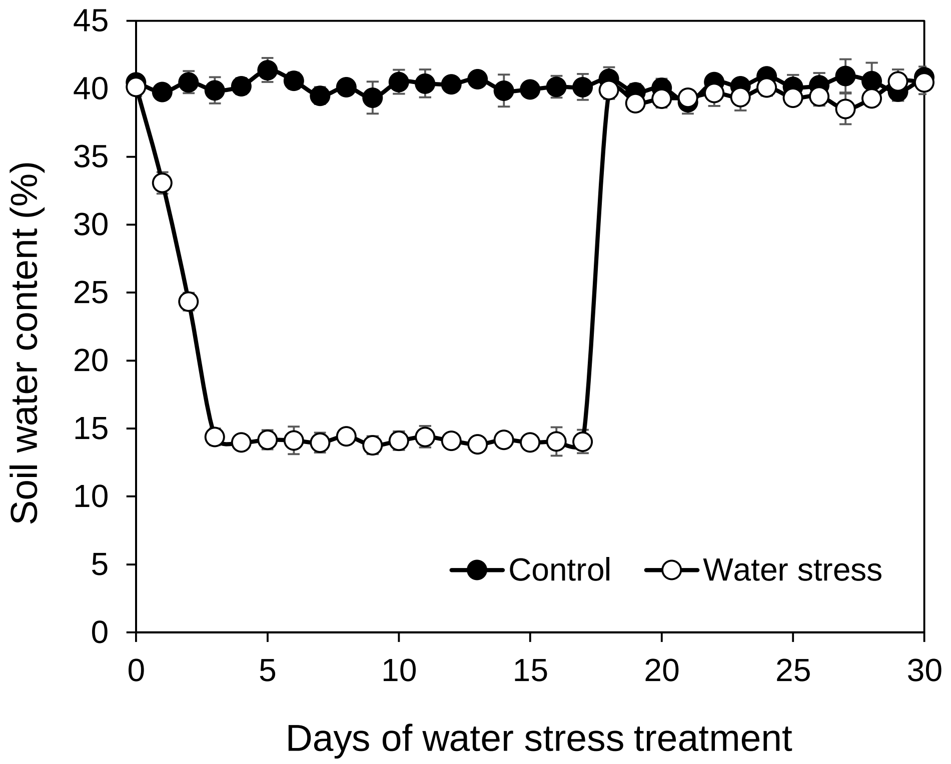

Figure S1 Changes in soil water content along with the treatment of water stress. Data are means ± SD on average of four barley genotypes.
